# The mental health toll among healthcare workers during the COVID-19 Pandemic in Malawi

**DOI:** 10.1038/s41598-024-61216-x

**Published:** 2024-05-06

**Authors:** Limbika Maliwichi, Fiskani Kondowe, Chilungamo Mmanga, Martina Mchenga, Jimmy Kainja, Simunye Nyamali, Yamikani Ndasauka

**Affiliations:** 1 University of Malawi, Zomba, Malawi; 2 Kamuzu University of Health Sciences, Lilongwe, Malawi; 3 University of Cape Town, Cape Town, South Africa

**Keywords:** Depression, Anxiety, Post-traumatic stress disorder, COVID-19, Healthcare workers, Mixed methods, Stress process model, Malawi, Public health, Health care, Occupational health

## Abstract

The COVID-19 pandemic has affected the mental health of healthcare workers worldwide, with frontline personnel experiencing heightened rates of depression, anxiety, and posttraumatic stress. This mixed-methods study aimed to assess the mental health toll of COVID-19 on healthcare workers in Malawi. A cross-sectional survey utilising the Generalized Anxiety Disorder (GAD-7), Patient Health Questionnaire (PHQ-9), and Primary Care PTSD Screen for DSM-5 (PC-PTSD-5) was conducted among 109 frontline healthcare workers. Additionally, in-depth interviews were conducted with 16 healthcare workers to explore their experiences and challenges during the pandemic. The results indicated a high prevalence of COVID-19-related depression (31%; CI [23, 41]), anxiety (30%; CI [22, 40]), and PTSD (25%; CI [17, 34]) among participants. Regression analysis revealed significantly higher rates of depression, anxiety, and PTSD among healthcare workers in city referral hospitals compared to district hospitals. Qualitative findings highlighted the emotional distress, impact on work and personal life, and experiences of stigma and discrimination faced by healthcare workers. The stress process model provided a valuable framework for understanding the relationship among pandemic-related stressors, coping resources, and mental health outcomes. The findings underscore the urgent need for interventions and support systems to mitigate the mental health impact of COVID-19 on frontline healthcare workers in Malawi. Policymakers should prioritise the assessment and treatment of mental health problems among this critical workforce to maintain an effective pandemic response and build resilience for future crises.

## Introduction

This study aims to assess the mental health toll of COVID-19 on healthcare workers in Malawi. In March 2020, the World Health Organization (WHO) declared COVID-19 a global pandemic, given its rapid worldwide spread^1^. Malawi registered its first case of COVID-19 in March 2020. According to statistics from the World Health Organization^2^, Malawi has registered approximately 89,000 confirmed cases of COVID-19, resulting in 2,686 deaths. While these numbers are relatively low compared to many other countries, the pandemic nevertheless placed immense strain on Malawi's healthcare system, which was already struggling with limited resources and infrastructure before the crisis.

COVID-19 has affected mental health worldwide, with the general population and healthcare workers experiencing heightened rates of depression, anxiety and posttraumatic stress^3,4^. COVID-19-related mental health challenges in frontline health personnel could negatively impact healthcare provision, compounding the pandemic's strain on health systems. Pandemics can generate multifaceted stressors, including fear of infection, financial loss, work disruptions, and social isolation, which may adversely impact the population's mental health^3^.

One of the most striking findings across studies is the high prevalence of mental health problems among healthcare workers during the COVID-19 pandemic in LMICs. Systematic reviews and meta-analyses by Chen, et al.^5^ and Luo, et al.^6^ provide an overview of the magnitude of the issue, with pooled prevalence rates ranging from 31.6% to 43.6% for depression, 37.2% to 37.3% for anxiety, and 31.4% to 49.5% for post-traumatic stress disorder (PTSD) among healthcare workers in Africa and other LMICs. These rates are considerably higher than those reported in the general population, underscoring the disproportionate psychological burden experienced by frontline health personnel in resource-constrained settings.

Studies focused on specific countries within sub-Saharan Africa, such as Zimbabwe^7^, Malawi^8,9^, South Africa^10,11^, Nigeria^12,13^, and Uganda^14^, consistently report high levels of psychological distress, anxiety, depression, and PTSD among healthcare workers. For instance, Chingono, et al.^7^ found that over 53% of healthcare workers in Zimbabwe experienced moderate to severe psychological distress. At the same time, Erinoso, et al.^12^ reported high rates of anxiety (40%) and depression (40%) among frontline nurses in Nigeria. These findings are not limited to sub-Saharan Africa, as studies from other LMICs, such as Pakistan^15^ and Nepal^16^, have yielded similar results. The pervasive nature of the mental health impact across different regions highlights the global scale of the problem and the need for urgent action to support healthcare workers in resource-constrained settings.

Further, the high prevalence of mental health problems among healthcare workers can be attributed to a complex interplay of occupational and personal stressors. Studies identify several key occupational stressors that contribute to psychological distress, including increased workload, lack of personal protective equipment (PPE), fear of infection, and exposure to patient suffering and death. Qualitative studies provide valuable insights into the emotional toll of these occupational stressors on frontline health personnel. For example, Dawood, Tomita and Ramlall^11^ found that healthcare workers in South Africa's KwaZulu-Natal Province felt unheard and unsupported by their employers and the government, leading to feelings of burnout, anxiety, and depression. Similarly, Mahlangu, et al.^10^ reported that healthcare workers in South Africa experienced memories of traumatic experiences, such as witnessing patient deaths and fearing for their safety. In Nigeria, Kwaghe, et al.^13^ highlighted the role of stigma and lack of support from communities and employers in exacerbating the psychological impact of the pandemic on healthcare workers. Participants in this study reported experiencing fear, anxiety, and emotional distress due to the risk of infection, stigma, and inadequate support systems.

Personal stressors, such as concerns for the well-being of family members, have also emerged as significant contributors to mental health challenges among healthcare workers. Sia-Morenike, et al.^17^ found that frontline health personnel in Sierra Leone experienced anxiety and fear not only for their health but also for the safety of their loved ones. The challenges of balancing professional duties with personal and family responsibilities have added to the psychological burden healthcare workers face during the pandemic. The interplay of occupational and personal stressors is further complicated by the resource constraints and systemic challenges faced by healthcare systems in sub-Saharan Africa and other LMICs. Lack of adequate resources, such as PPE, medical equipment, and trained personnel, has increased workload and heightened stress among frontline health workers^17^. Furthermore, pre-existing issues such as underfunded health systems, limited mental health infrastructure, and social stigma associated with seeking psychological support have exacerbated the mental health impact of the pandemic on healthcare workers in these regions.

In addition, findings from studies underscore the urgent need for targeted interventions and support systems to mitigate the mental health impact of the COVID-19 pandemic on healthcare workers in sub-Saharan Africa and other LMICs. Moitra, et al.^18^ and Oyat, et al.^19^ emphasise the importance of providing stress management training, peer support, and access to psychological services to support healthcare workers' mental health. Munyenyembe and Chen^9^ and Oyat, et al.^19^ provide valuable insights into the coping strategies used by healthcare workers, such as seeking social support, engaging in leisure activities, and relying on faith. However, these studies also highlight the barriers to coping, including lack of resources and stigma associated with seeking mental health support. These findings suggest that interventions should focus not only on enhancing individual coping capacities but also on addressing the structural and systemic factors that contribute to psychological distress.

The current study employed a mixed-methods approach integrating quantitative screening instruments with qualitative interviews to address these knowledge gaps. Combining these methodologies allowed for assessing depression, anxiety and PTSD prevalence while also capturing nuanced insights into healthcare workers' pandemic experiences. The qualitative component was critical for understanding how Malawian healthcare workers made sense of the unprecedented crisis and its mental health ramifications. In-depth interviews explored healthcare workers' responses and perspectives on mental health within their socio-cultural context. This qualitative lens complemented quantitative findings, enabling a more comprehensive, contextually-grounded analysis. The stress process model provides a valuable framework for examining how pandemic-related stressors may impact mental health^20^. This model posits that stressors arising from social circumstances can precipitate psychological distress, with coping resources potentially mitigating adverse effects. The model also recognises both the direct impacts of stressors and indirect effects via the erosion of coping mechanisms. COVID-19 represents an acute, society-wide stressor that has dramatically transformed healthcare workers' occupational and social realities. Findings can inform interventions to support this critical workforce and guide mental health responses to COVID-19 in similar contexts. More broadly, this study highlights the need to invest in mental health infrastructure and prioritise psychological support within the pandemic preparedness in LMICs.

## Materials and methods

### The stress process model

There are several models that researchers may consider using in COVID-19 research to explore the interplay of stress, coping, and resilience in individuals and communities. Some of these models include the transactional model of stress and coping, which emphasises the dynamic relationship between individuals and their environment during the stress-coping process. The biopsychosocial model considers biological, psychological, and social factors influencing health and well-being. The social-ecological model examines how the environment multiple levels of their environment influence individuals. The resilience model emphasises individuals' capacity to adapt and bounce back from adversity. Each model offers unique insights and perspectives that can contribute to a comprehensive understanding of the psychological and social impacts of the pandemic.

This study utilises the stress process model because it offers a comprehensive framework for studying the interplay among stressors, coping mechanisms, and outcomes in COVID-19. The stress process model^20^ provides a valuable framework for examining how exposure to pandemic-related stressors may impact mental health outcomes in healthcare workers. This model posits that stressors arise from individuals' social circumstances and roles. Prolonged or intense stressors can manifest mental health symptoms through processes like the erosion of positive coping resources. Researchers have applied this model to examine chronic stressors' impact on doctoral students' mental health outcomes^21^. The study revealed academic stressors to be the strongest predictors of burnout, while family and monetary stressors were most closely associated with depression. It also found that the relationship between stress and burnout was partially mediated by mastery and advisor support, whereas the relationship between stress and depression was partially mediated by mastery. Likewise, Gilster^22^ used the stress process model to investigate the neighbourhood stress process, particularly exploring racial and ethnic variations in the associations between neighbourhood stressors, mastery, and depressive symptoms within a multi-ethnic sample population. The study found that mastery partially mediates the relationship between perceived and observed neighbourhood stressors and depressive symptoms but does not provide a buffering effect against neighbourhood stressors.

In this study, the COVID-19 pandemic represents an acute and enduring stressor event that has fundamentally altered the social circumstances and occupational roles of frontline healthcare workers in Malawi and other LMICs. Specific pandemic-related stressors include heightened risk of infection, resource shortages, heavy workloads, loss of patients, and moral distress. The stress process model would suggest that these stressors can directly impact healthcare workers' mental health. Guided by the stress process model, this study analyses the relationships between pandemic stressor exposure, availability of coping resources, and mental health to determine predictive pathways. The framework recognises the pandemic's role as a significant stressor while accounting for individual and systemic protective factors that may confer resilience despite circumstances.

### Study design, setting and participants

This cross-sectional study was conducted from May to June 2021 and utilised a mixed methods approach by combining quantitative and qualitative methods. The study was conducted in 4 districts (Blantyre, Mangochi, Lilongwe and Karonga) from the four regions of Malawi. We selected these districts because two are cities with large central hospitals handling severe and most COVID-19 cases (Lilongwe and Blantyre), one is a Lake District (Mangochi), which may be affected by dropped levels of tourism due to COVID-19, and finally a boarder district (Karonga) which is a possible entry point for imported COVID-19 cases. For qualitative data, we targeted frontline health workers with first-hand knowledge of the COVID-19 situation in hospitals and supervisors/managers who are believed to have a better idea of the situation in the four hospitals. Key Informant Interviews (KIIs) were conducted with four chief nursing officers or matrons. 12 In-depth Interviews (IDIs) were conducted with frontline health workers, three from each of the four hospitals. The data collected provided in-depth information to address the study objectives. Those who participated in the qualitative interviews did not participate in the quantitative survey and were purposively selected from hospital health workers on the data collection days. The sample of n = 119 was estimated using the sample size calculation formula for an unknown total population^23^. We used probability proportional to size (PPS) so that each hospital contributes a proportional sample depending on the number of health workers. Convenience sampling was used to select the respondents. However, 109 frontline clinicians and nurses from the four hospitals were interviewed to assess the prevalence of depression, anxiety and PTSD, representing a 91.5% response rate.

### Measures

Interview guides and interviewer-administered questionnaires were used to collect qualitative and quantitative data. With practical experience and expertise in the mental health field for content and construct validity, the research team developed and reviewed the interview guide, which explored the experiences of frontline health workers. This guide was administered in English and Chichewa, and the interviews were conducted by research supervisors who had experience conducting key informant interviews. To assess the mental health status of participants, the study utilised the Generalized Anxiety Disorder (GAD-7), Patient Health Questionnaire (PHQ-9) and the Primary Care Post Traumatic Symptom Disorder Screen for DSM-5 (PC-PTSD-5). The three questionnaires have been used in various populations as brief screening measures for depression and anxiety^24,25^. Additionally, the PHQ-9 has been used before in Malawi^26^. PHQ-9 has nine items which measure depressive symptoms in the past two weeks on a scale of 1–27. The GAD-7 measures anxiety symptoms in the past two weeks and has seven questions with options ranging from 0 (not at all) to 3 (nearly every day) with an overall scale of 0–21. The PC-PTSD-5 is a 5-item questionnaire that measures posttraumatic symptoms in the past month on a scale of 1–5. The higher the scores, the more severe the symptoms were on all three questionnaires.

### Data analysis

Quantitative data were analysed using STATA version 14. Scores were calculated for depression, anxiety and PTSD. Depression was measured by PHQ-9 on a scale of 0–27 and was classified into the following five groups: minimal (1–4), mild (5–9), moderate (10–14), moderately severe (15–19), and severe (20–27). The GAD-7 anxiety questionnaire had an overall score range of 0–21, which was classified into the following four categories: no anxiety or minimal (< 5), mild anxiety (5–9), moderate anxiety (10–14), and severe anxiety (> 15). PTSD was measured using a PC-PTSD-5 questionnaire on a scale of 1–5, with a cut-off of 3 points, i.e. PTSD was reported as observed for scores > 2. Binary outcomes are reported as depressed (score < 5) or not depressed (score >  = 5), anxious (score < 5) or not anxious (score >  = 5), PTSD (score < 3) or no PTSD (score >  = 3). We tested the differences in proportions within population characteristics and the outcomes of interest using the Mc-Nemas chi-square test for proportions. We used binary logistic regression to explore factors associated with the three binary outcomes. All tests were conducted at a 95% confidence level.

Qualitative data were analysed using thematic analysis from a constructivist perspective. The study utilised the six phases of thematic analysis. The first step was data familiarisation, which involved reading and re-reading all KIIs and IDIs transcripts. Secondly, we generated initial codes from the data set identifying essential features relevant to answering the research question. Thirdly, we collated initial codes to identify significant broader patterns of meaning (potential themes) and review the viability of each theme. Fourth, we examined the themes to check if they addressed the research question. Some themes were split, combined, or discarded. Fifth, we defined and named the themes and worked out the scope and focus of each theme. Finally, we drafted the paper using triangulated quantitative and qualitative data, paying attention to context and the existing literature. The draft manuscript was circulated to the team members for their final review before consolidating all the revisions and submitting the finalised paper.

### Ethics approval and consent to participate

The study fully adhered to ethical standards expressed in the Declaration of Helsinki. Before the commencement of the study, relevant authorisation and approval were sought from the University of Malawi Research Ethics Committee (UNIMAREC) (No. P/03/21/53), Mangochi, Blantyre, Lilongwe and Karonga District Health Officers (DHOs) and hospital directors. Participants who were above eighteen years old and agreed to participate in the study provided written informed consent to participate.

## Results

### Demographic characteristics

Table 1 shows the basic demographic details of the study participants.

**Table 1:** Demographic characteristics.

Characteristic	N (%)
District	
Lilongwe	34 (31)
Blantyre	50 (46)
Mangochi	15 (14)
Karonga	10 (9)
Hospital type	
Referral	84 (77)
District	25 (23)
Gender	
Male	46(42)
Female	62(57)
Position	
Nurse	37 (34)
Clinician	30 (28)
Unknown	42 (39)
Age in years	
< 30	48 (44)
30–39	39 (36)
> 39	17 (16)
Total	109(100)

According to Table 1, most study participants were female, 57% (n = 62), while males comprised 42% (n = 46). The age range of participants was from 21 to 65 years old, with a mean age of 33 (SD = 7.9) years old, and the majority aged below 40 years old, 80% (n = 87). Almost half of the participants were from Blantyre, 46% (n = 50). Most respondents, 77% (n = 84), were from city referral hospitals of Blantyre and Lilongwe, while only 23% (n = 25) were from the district hospitals of Mangochi and Karonga).

### Prevalence of COVID-19-related depression, anxiety and PTSD

Table 2. shows the point prevalence of COVID-19-related depression segregated by demographic factors.

**Table 2:** Prevalence of COVID-19-related depression, anxiety, and PTSD.

Demographic Factors	Depression	Anxiety	PTSD
	Proportion with outcome n(P) [CI]		
	34(31)[23, 41]	33(30) [22, 40]	27 (25) [17, 34]
	Proportion with outcome n (%) [*P*-value]		
District			
Lilongwe	10 (29)	15 (44)	12 (35)
Blantyre	20 (40)	17 (34)	13 (26)
Mangochi	3 (20)	0 (0)	0 (0)
Karonga	1 (10)	1 (10)	2 (20)
Hospital type	[0.061]	[0.001]	[0.026]
Referral	30 (36)	32 (38)	2 (8)
District	4 (16)	1 (1)	25 (30)
Gender	[0.06]	[0.196]	[0.16]
Male	10 (21)	11 (24)	8 (17)
Female	24 (39)	22 (35)	18 (29)
Position			
Nurse	11 (30)	13 (35)	11 (30)
Clinician	13 (43)	13 (43)	9 (30)
Missing	10 (24)	7 (17)	7 (17)
Age in years			
< 30	15 (31)	19 (40)	10 (21)
30–39	11 (28)	9 (23)	10 (26)
> 39	6 (35)	4 (24)	3 (18)
Total	34 (31)	33 30)	27 (25)

**Figure 1:**
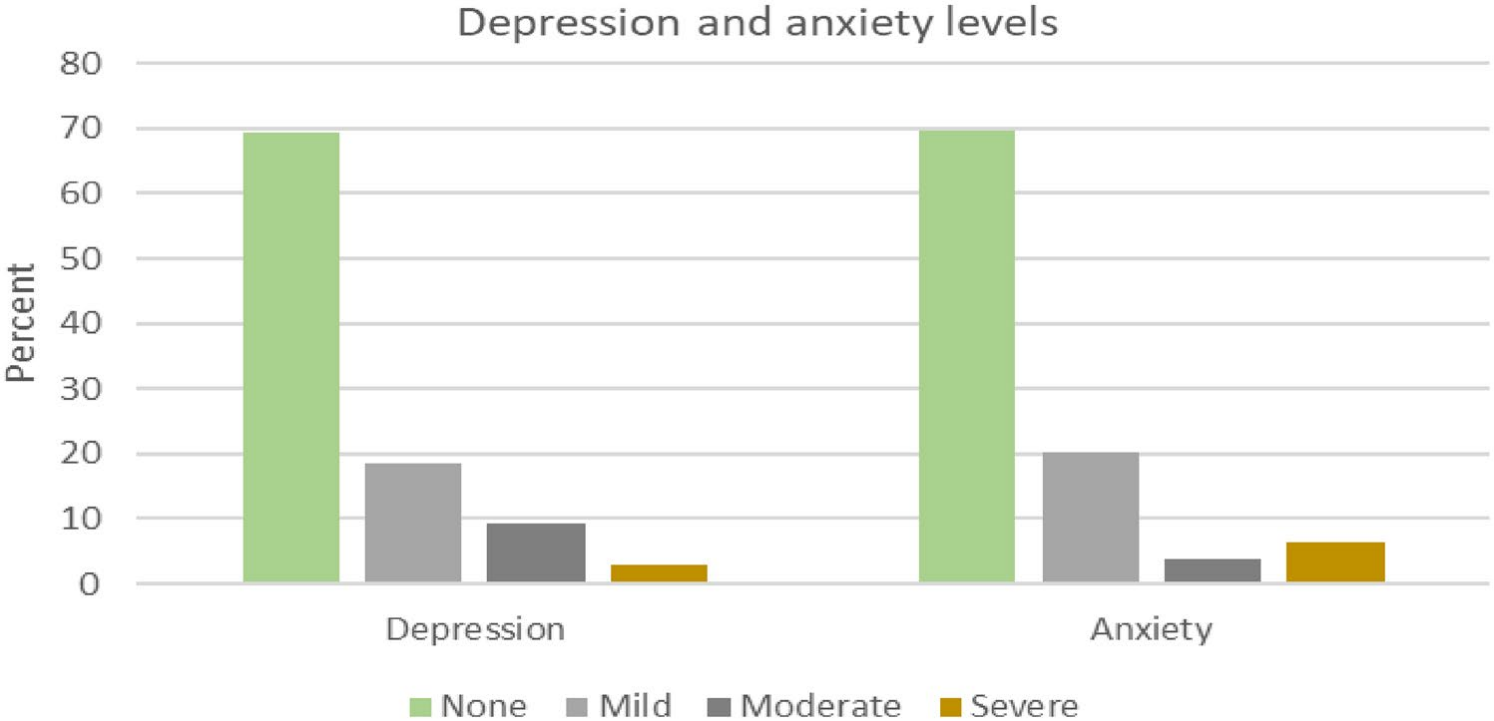
Levels of depression and anxiety for sampled health care workers.

The results of this study (Table 2) indicated an overall high prevalence of COVID-19-related depression (31%; CI [23, 41]), anxiety (30%; CI [22, 40]) and PTSD (25%; CI [17, 34]) among health care workers. However, most respondents had mild depression (18.4%) or anxiety (20.2%), as highlighted in Fig. 1.

A high proportion of depression was observed in Blantyre (n = 20, 40%) and among female health workers (n = 24, 39%). On the other hand, a high proportion of anxiety was observed in Lilongwe (n = 15, 44%) and among female health workers (n = 22, 35%). Furthermore, a high proportion of PTSD incidence was observed in Lilongwe (n = 12, 35%) and among female health workers (n = 18, 29%).

Despite the observed differences in depression, anxiety and PTSD prevalence for different variable groups, the regression results reveal that these differences are not statistically significant (Table 3).

**Table 3:** Demographic factors associated with depression, anxiety and PTSD among health workers.

Variables	Depression	Anxiety	PTSD
	OR [95% CI]		
Gender			
Male (Ref)			
Female	2.3 [0.91, 6.02]	1.41 [0.54, 3.69]	1.95 [0.68, 5.59]
Age			
30–39 years (Ref)			
30–39 years	1.08 [0.40, 2.94]	0.57 [0.20, 1.59]	1.65 [0.56, 4.84]
> 39 years	1.20 [0.34, 4.18]	0.41 [0.11, 1.64]	0.73 [0.16, 3.29]
Hospital type			
District (Ref)			
Referral	1.8 [0.42, 8.02]	8.63 [0.95, 78.16]	2.70 [0.44, 16.74]
Positions			
Nurse [Ref]			
Clinician	2.21 [0.75, 6.53]	1.19 [0.41, 3.44]	1.38 [0.44, 4.34]
Unknown	1.07 [0.31, 3.66]	0.59 [0.17, 2.05]	0.66 [0.17, 2.61]

Multivariable logistic regression results for depression, anxiety and PTSD among health workers.

*Significant odds ratio compared to the indicated reference (ref) group.

The qualitative data analysis revealed three main themes that encapsulate the experiences and challenges faced by healthcare workers in Malawi during the COVID-19 pandemic: 1) Mental health symptoms and emotional distress, 2) Impact on work and personal life, and 3) Stigma and discrimination.

### Mental health symptoms and emotional distress

The healthcare workers across the four districts reported experiencing a wide range of concerning mental health symptoms related to depression, anxiety, and posttraumatic stress disorder (PTSD). Regarding depressive symptoms, many workers described persistent sadness, hopelessness, loss of interest in everyday activities, fatigue, worthlessness or guilt, difficulty concentrating, and recurrent thoughts of death or suicide. The healthcare workers highlighted excessive worry, restlessness, difficulty relaxing, irritability, muscle tension, sleep disturbances, and panic attacks as some of the anxiety-related symptoms they had been experiencing. Additionally, the workers reported PTSD-associated symptoms, including unwanted distressing memories of treating COVID-19 patients and emotional and physical reactions when reminded of traumatic pandemic experiences. This avoidance of people or situations triggers recollections of trauma, hypervigilance, and exaggerated startle response. *"Memories of the dead from Covid-19 haunted most of us. Most of the night, I failed to sleep. Some health workers reported the same to me. They would come for help, but I could only give them sleeping pills"* (Matron, Karonga). *"Sometimes, you just wish you could just shut down. Just forget everything. It was too much for us; too much sadness" (Matron, Lilongwe)*.

According to the healthcare workers' accounts, these mental health symptoms had persisted for several months since the onset of the COVID-19 pandemic and significantly impacted their daily functioning and quality of life. Some workers reported being unable to get out of bed in the morning due to depression, while others described intense anxiety preventing them from being able to focus at work. The intrusive re-experiencing symptoms of PTSD were causing some workers substantial distress. Several workers tearfully recalled traumatic events such as holding the hands of dying COVID patients. These traumatic memories now haunted the healthcare workers, causing sleep disturbances and emotional anguish. In addition, traumatic experiences related to COVID-19 patient care also led to PTSD symptoms, such as intrusive memories and emotional numbing. A nurse in Lilongwe recounted, "*I can still hear the sound of the ventilator alarms and see the faces of the patients who passed away. It is like a constant reminder of the tragedy we witnessed.*"

In addition to the associated symptoms, healthcare workers also described profound emotional distress from their experiences of rapid patient deterioration and death in COVID-19 cases. As one clinician from Mangochi district poignantly recounted: *"Most people say medical personnel are used to that, but with that disease, it is not easy to see people who were okay suddenly got the disease, it gets worse, and suddenly they die; it was too much to accept it." (Clinician, Mangochi).* Further, anxiety symptoms were also prevalent, with healthcare workers mentioning excessive worry, restlessness, and sleep disturbances. A clinician in Karonga remarked, "*I would lie awake at night, my mind racing with thoughts of all the patients I could not save and the constant fear of bringing the virus home to my family.*"

These clinicians’ accounts provide insight into the trauma and moral injury inflicted on healthcare workers by the stark severity and frequent fatalities of COVID-19. It exemplifies the psychological impact of experiencing rapid patient declines and deaths first-hand, sometimes without adequate resources to prevent grievous outcomes. The sentiment expressed by this frontline worker in Mangochi district was similarly echoed by many others who struggled to come to terms with the sudden loss of life amongst their patients. One nurse from Karonga shared, "*I felt so overwhelmed and helpless seeing patients deteriorate despite our best efforts. It was like a never-ending nightmare*." These experiences likely contributed to the substantial burden of depressive, anxiety, and PTSD symptoms endorsed by the healthcare workers amid Malawi's COVID-19 response.

### Impact on work and personal life

Participants reported that frontline health workers were overwhelmed with work due to an increase in patient numbers and a reduction in the number of health workers. However, in Mangochi, respondents reported a general decrease in patient numbers, approximately from 60 patients per day to 30 per day; this did not translate to a lower workload since there was also a reduction in health workers for various reasons such as unexplained absenteeism, sickness and transfers. One nurse from Mangochi shared, "*We were stretched thin, working endless hours with little rest. It was physically and emotionally draining."* The same applied to Karonga, where healthcare workers were expected to work continuously for two weeks, followed by a week's holiday. This work overload may have contributed to "burnout", as reported by most participants. *"The workload has increased, as you know. The pandemic, especially the second wave, hit us hard, so the workload just increased suddenly" (Nurse, Karonga).*

Respondents also reported reduced face-to-face interaction with fellow workers, mainly due to new work protocols. COVID-19 measures, such as social distancing and the frequent use of Personal Protective Equipment (PPE, reduced their sense of connectedness. In addition, work output was negatively affected; participants attributed this to increased absenteeism, attrition and application of leave days, which was attributed to workers' fear of contracting COVID-19 at the workplace. Participants reported more panic and fear during the first wave than in the second since the healthcare workers might have developed strategies and coping mechanisms. *"… so, there was less panic and fear in responding to the second wave because we had now learnt what we should do” (Matron, Lilongwe).* Further, the fear of contracting the virus and infecting loved ones also took a toll on healthcare workers' family dynamics. "*I had to isolate myself from my family for months, unable to hug my children or comfort them when they needed me the most. It was a painful sacrifice*" (Clinician, Blantyre).

The COVID-19 pandemic also disrupted healthcare workers' family relationships and social connections in numerous ways. Many participants described the constant terror of spreading the virus to loved ones. This led some workers to agonise to isolate themselves entirely from family to avoid potential transmission. One matron from Lilongwe explained: "*There was a time I thought I should send my wife and children home so that I should remain alone*." Sacrificing these crucial family bonds during an already stressful period exacted deep emotional pain, leaving workers feeling alone when support was most needed. Even for those who did not entirely self-isolate, the spectre of viral transmission strained family dynamics. Workers reported feeling unable to embrace loved ones, attend important events like weddings and funerals, or find comfort in casual physical affection. Families are vital for mental health; this constant stress and distance in relationships took a substantial psychological toll. Further, healthcare workers also expressed grief over the loss of colleagues and the impact on team morale. "*Losing our coworkers to COVID-19 was devastating. It felt like a part of our family was gone, and the void they left behind was immense*" (Nurse, Karonga).

### Stigma and discrimination

Healthcare workers faced significant stigma and discrimination from their communities due to their occupation. Many reported instances of social isolation, harassment, and even violence. One nurse from Mangochi recounted, "*People would yell at me on the street, calling me a virus carrier. They would cross the road to avoid passing by me as if I were a walking biohazard.*" The stigma also extended to their living situations, with some healthcare workers being evicted from their homes by landlords who feared they would spread the virus. "*I was kicked out of my rental apartment because the landlord found out I worked in a COVID-19 unit. I had nowhere to go and felt utterly betrayed*" (Nurse, Lilongwe).

Participants described pervasive stigma from the community that damaged their social lives and sense of self. Many healthcare workers felt ostracised by the communities they sought to serve. Friends and neighbours avoided interacting with them in public due to transmission fears. This social stigma made healthcare workers increasingly isolated from previous social supports. For some healthcare workers, the stigma escalated to threats of physical violence. Multiple accounts emerged of community members throwing rocks at visiting health workers or barricading them from entering villages to administer vaccines. Workers feared that misinformation linking them to virus transmission had provoked this aggression. The social rejection, housing discrimination, and violence described by participants illustrate the heavy emotional toll of stigma during public health crises. Health workers felt hurt and afraid in the very communities they sacrificed to protect. This mistreatment exacerbated an already isolating and stressful pandemic working environment.

Many healthcare workers experienced self-stigma and were reluctant to seek mental health support despite the availability of some psychosocial support services. They feared further stigmatisation from colleagues who might perceive them as weak or incapable of handling their job responsibilities. A clinician in Blantyre admitted, "*I was afraid to admit I needed mental health support. I did not want my colleagues to think I was weak or incapable of handling my job*." In addition to the fear of stigmatisation, many workers were unaware of the existence of in-house counselling or support services. Heavy workloads also left them little time to pursue such services, even when they knew their availability.

## Discussion

This study assessed the toll of COVID-19 on the mental health of frontline health workers and explored the experiences of these health workers in Malawi. The results showed relatively higher depressive, anxiety and PTSD symptoms due to COVID-19. This may be due to increased levels of work and fear for self and family. Whilst healthcare workers enjoyed a heroic reception in some parts of the world, they were discriminated against in Malawi. This added a toll on the mental health of healthcare workers.

On depression, a recent meta-analysis of a combined total of 33,062 participants showed a depression pooled prevalence rate of 22·8%^27^. Another review of 25 systematic reviews on primary studies with healthcare workers and other vulnerable groups showed that depression prevalence rates range from 20 to 51%^28^. This study found a depression prevalence rate of 31%. This higher rate may partly be explained by the unique challenges faced by the health workers in Malawi during the COVID-19 pandemic. One of the significant identified challenges highlighted in this study was the availability of limited resources in hospitals. These resources include Personal Protective Equipment (PPEs), syringes and blood bags that are crucial for healthcare workers' ability to effectively assist patients while preventing the transmission of diseases, including COVID-19. Inadequate human resources due to staff turnover and attrition leading to long working hours of the available health workers was another unique identified challenge.

Additionally, reduced face-to-face interaction with fellow workers, mainly due to new work protocols, decreased their sense of connectedness. Finally, stigmatisation and discrimination of health workers by the community resulted in reduced interaction between health workers and the community, thereby enhancing depressive symptoms. Likewise, stigmatising health workers experiencing mental health problems by their fellow health workers/colleagues may have negatively affected help-seeking behaviours, thereby worsening their condition^29^.

The high prevalence rate of COVID-19-related anxiety among health workers found in this study is slightly higher than the one reported in another recent study on nurses in Malawi, which found a prevalence rate of 25.5% on 26 nurses using the CAS^8^. However, the same study reported a slightly higher prevalence of COVID-19-related anxiety among hospital nurses (36.2%, n = 21). The slight differences may be attributed to differences in study design, anxiety measurement tools, cadre of health workers and time of data collection about COVID-19 waves. We collected our data in May 2021, four months after the pick of the second wave, when health workers were more confident in handling COVID-19 cases and were more aware of myths and misconceptions about the mode of COVID-19 transmission. These results contrast the low-pooled anxiety prevalence of 23·2%^5,30^.

Another review of 25 systematic reviews on primary studies with healthcare workers and other vulnerable groups showed that anxiety prevalence rates range from 12 to 45%^29^. The anxiety symptoms experienced by the healthcare workers in Malawi may be explained by their fear of contracting COVID-19 at the workplace. Likewise, the healthcare workers were also afraid of going home after work, fearing the possibility of transmitting the virus to their families. This explains the increased absenteeism, attrition and application of leave days. However, there was more panic and fear during the first wave compared to the second wave since the healthcare workers might have better understood the COVID-19 mode of transmission and prevention and developed better-coping mechanisms and strategies.

The PTSD prevalence rate of 24% found in this study is comparable to the results of other studies. A recent review of 25 systematic reviews on primary studies with healthcare workers and other vulnerable groups showed PTSD prevalence rates range from 19 to 51%^29^. The PTSD symptoms may be attributed to the health workers witnessing sudden deaths of COVID-19 patients. Even though the study did not find any case of healthcare workers with PTSD in Mangochi, there is evidence that some healthcare workers are experiencing PTSD symptoms, as reported by key informants.

Another significant finding of this study was that there are more healthcare workers with depression, anxiety and PTSD in city referral hospitals compared to district hospitals. These results may be attributed to several factors, including a higher workload for health workers due to increased COVID-19 patients in city referral hospitals than in district hospitals. As the most significant urban centres in the country, Blantyre and Lilongwe have higher population densities than rural districts. This demographic factor likely contributed to more COVID-19 cases in these cities, as crowded living conditions can facilitate the spread of the virus. Consequently, the referral hospitals in Blantyre and Lilongwe experienced a more significant influx of COVID-19 patients than district hospitals, placing a considerable burden on the healthcare workers in these facilities. These facilities also managed the most severe COVID-19 cases across Malawi. This concentration of critically ill patients likely exposed healthcare workers in these facilities to heightened levels of stress, trauma, and moral distress. Witnessing high rates of patient deterioration and death, coupled with the need to make difficult triage decisions amidst resource constraints, may have exacerbated the psychological toll on frontline staff in these urban referral hospitals. In contrast, some district hospitals, such as Mangochi, reported a significant decrease in patient numbers across all departments during the pandemic. This trend could be attributed to reduced health-seeking behaviour due to fear of contracting COVID-19 and the diversion of healthcare resources towards the pandemic response. While a decline in patient volume may have alleviated some workload pressures, it also raises concerns about potential unmet health needs and delayed care for non-COVID conditions in these rural districts.

Some of the identified challenges experienced by healthcare workers during the pandemic included limited resources such as PPEs, syringes and blood bags, inadequate human resources, long working hours, lack of awareness of available services such as counselling, staff turnover and attrition^31^. In addition, re-assigning most healthcare workers to work in COVID-19 centres created gaps in other departments that overwhelmed the remaining staff. Despite the several challenges faced due to the COVID-19 pandemic, respondents identified some opportunities that COVID-19 brought to mental health in Malawi. Firstly, more health workers were recruited in response to COVID-19. For example, a healthcare worker in Karonga reported receiving additional nurses during the first and second waves of the pandemic. Secondly, the government directed funds towards mental health and recruited more mental health workers, such as psychosocial counsellors. This is the first time in Malawi that the government has employed this particular cadre of health workers. Thirdly, public and private sectors offered several trainings to health workers, including those focusing on COVID-19 safety and management. Finally, both formal and informal psychosocial interventions were available. However, the utilisation of such interventions by health workers could have been more extensive. This was mainly attributed to fear of stigmatisation and lack of knowledge of such in-house services.

Even before the COVID-19 pandemic, healthcare workers in Malawi faced significant psychosocial challenges and resource constraints that likely heightened their vulnerability to the mental health consequences of the crisis. A 2018 study found that 62% of Malawian healthcare workers reported burnout, with emotional exhaustion being a shared experience^32^. Inadequate staffing, heavy workloads, lack of essential medical supplies, and low salaries contribute to healthcare worker burnout and job dissatisfaction^32^. These pre-existing issues, coupled with Malawi's critical shortage of healthcare personnel, created a context of chronic occupational stress and under-resourcing. The country has long struggled to train and retain an adequate health workforce, with a deficient number of physicians and nursing/midwifery personnel per 1,000 population^33^. This shortage has been compounded by the uneven distribution of health workers across urban and rural areas, high rates of attrition, and the burden of HIV/AIDS^34^. Consequently, when the COVID-19 pandemic struck, Malawian healthcare workers were already grappling with intense workloads, resource deficits, and psychosocial strain, potentially amplifying the mental health repercussions of pandemic-related stressors documented in this study.

The quantitative and qualitative results align closely with the stress process model's premise that exposure to stressors emerging from one's social circumstances can precipitate mental health symptoms without protective resources. The pandemic undoubtedly represented an abrupt stressor event that dramatically transformed participants' work environments and responsibilities. Quantitatively, high rates of depressive, anxiety and PTSD symptoms were documented. While not conclusively diagnostic, these screening results suggest a substantial burden of psychological distress consistent with other studies of frontline workers during COVID-19. Qualitative accounts provided vivid examples of pandemic-related stressors frequently cited in stress process model research as risk factors for poor mental health, including lack of personal protective equipment, heavy workloads, loss of co-workers and patients to illness, and the constant risk of infection. Witnessing the sheer human toll exacted by the virus profoundly impacted participants.

The stress process model recognises both the direct effects of stressors and indirect impacts via the erosion of positive coping resources. Participants described isolation and how fear of infecting loved ones kept them from family and social gatherings that previously provided support. The stigmatisation of healthcare workers also emerged as a prominent theme, aligning with literature on mental illness stigma as a barrier to help-seeking. 

### Study limitation

One of the main limitations of this study is the use of convenience sampling, which may limit the generalisability of the findings to the broader population of healthcare workers in Malawi. Convenience sampling relies on recruiting participants who are easily accessible and available. This non-probability sampling method can introduce bias, as the sample may not accurately reflect the characteristics and experiences of the entire healthcare workforce. Additionally, the study's cross-sectional design provides only a snapshot of the mental health status of healthcare workers at a single point in time. This approach does not allow for examining causal relationships or changes in mental health outcomes throughout the pandemic. Furthermore, the study relied on self-reported measures of mental health symptoms, which may be subject to social desirability bias or recall bias. Healthcare workers may have underreported their symptoms due to stigma or fear of professional consequences, or they may have had difficulty accurately recalling their experiences and emotions during the height of the pandemic. In addition, the study collected data from 109 out of 119 participants. This may affect the representativeness of the findings.

## Conclusion

This mixed-methods study provides insights into the toll of the COVID-19 pandemic on mental health outcomes amongst frontline healthcare workers in Malawi. Utilising validated screening tools and in-depth interviews, the study revealed depression, anxiety, and posttraumatic stress disorder symptoms among clinicians and nurses across multiple districts. Qualitative findings contextualised these results, with participants recounting traumatic experiences of resource shortages, excessive workloads, stigmatisation and witnessing frequent patient deaths. These results align closely with the stress process model, emphasising the hazardous effects of acute stressor events and potential moderating resources. As Malawi continues battling COVID-19, implementing policies to support healthcare workers' mental health and capacity must be an urgent priority. Bolstering counselling services, reducing stigma, implementing self-care practices and peer support groups and allowing brief recuperative breaks could help strengthen coping. More broadly, this study exemplifies the pressing need for more investment in mental healthcare in Malawi and similar low-resource health systems. Protecting the mental well-being of healthcare workers through proactive interventions will be essential to maintaining an effective pandemic response and building resilience for future crises.

Based on the findings of this study, there are several recommendations that the researchers and governments of low-resource settings like Malawi can consider to better prepare for and mitigate the mental health toll on healthcare workers during public health crises like the COVID-19 pandemic:

1. Integrate mental health support into emergency response plans: Governments should proactively include mental health considerations and interventions as a core component of their emergency preparedness and response strategies. This includes allocating dedicated resources for mental health services, training healthcare workers in psychological first aid, and establishing clear protocols for accessing support.
2. Strengthen mental health infrastructure and workforce: Investing in developing mental health infrastructure and expanding the mental health workforce is crucial to ensure adequate capacity to meet the increased demand for services during crises. This may involve training more mental health professionals, integrating mental health into primary care, and leveraging digital platforms to improve access to care.
3. Governments should prioritise the well-being of frontline healthcare workers. They should recognise the unique challenges and stressors faced by healthcare workers during public health emergencies and prioritise their well-being. This includes ensuring adequate PPE provision, implementing reasonable work hours and rotations, and offering tailored mental health support services, such as counselling and peer support groups.
4. Address stigma and promote help-seeking behaviours: Governments should actively work to reduce mental health stigma and encourage healthcare workers to seek support when needed. This can be achieved through public awareness campaigns, education and training initiatives, and fostering a culture of openness and compassion within healthcare settings.
5. Conduct further research and monitoring: Governments should support ongoing research to better understand the mental health impacts of public health emergencies on healthcare workers and evaluate the effectiveness of interventions. Regular monitoring and assessment of mental health outcomes can inform policy decisions and guide the allocation of resources.

## Data Availability

Data for this report is only available upon request. To request the data, contact the corresponding author at yndasauka@unima.ac.mw.
